# Geographic distribution and prevalence of human echinococcosis at the township level in the Tibet Autonomous Region

**DOI:** 10.1186/s40249-022-00933-9

**Published:** 2022-01-21

**Authors:** Liying Wang, Gongsang Quzhen, Min Qin, Zehang Liu, Huasheng Pang, Roger Frutos, Laurent Gavotte

**Affiliations:** 1 National Institute of Parasitic Diseases, Chinese Centre for Disease Control and Prevention (Chinese Centre for Tropical Diseases Research); NHC Key Laboratory of Parasite and Vector Biology, WHO Collaborating Centre for Tropical Diseases, National Centre for International Research On Tropical Diseases, Shanghai, 200025 People’s Republic of China; 2 Cirad, UMR 17, Intertryp, Montpellier, 34398 France; 3 Espace-Dev, University of Montpellier, Montpellier, 34000 France; 4 Tibet Center for Disease Control and Prevention, NHC Key Laboratory of Echinococcosis Prevention and Control, Lhasa, 850000 China

**Keywords:** Human echinococcosis, Prevalence, Geographic distribution, Tibet Autonomous Region, China

## Abstract

**Background:**

Echinococcosis, a zoonotic parasitic disease, is caused by larval stages of cestodes in the *Echinococcus* genus. Echinococcosis is highly prevalent in ten provinces/autonomous regions of western and northern China. In 2016, an epidemiological survey of Tibet Autonomous Region (TAR) revealed that the prevalence of human echinococcosis was 1.66%, which was much higher than the average prevalence in China (0.24%). Therefore, to improve on the current prevention and control measures, it is important to understand the prevalence and spatial distribution characteristics of human echinococcosis at the township level in TAR.

**Methods:**

Data for echinococcosis cases in 2018 were obtained from the annual report system of echinococcosis of Tibet Center for Disease Control and Prevention. Diagnosis had been performed via B-ultrasonography. The epidemic status of echinococcosis in all townships in TAR was classified according to the relevant standards of population prevalence indices as defined in the national technical plan for echinococcosis control. Spatial scan statistics were performed to establish the geographical townships that were most at risk of echinococcosis.

**Results:**

In 2018, a total of 16,009 echinococcosis cases, whose prevalence was 0.53%, were recorded in 74 endemic counties in TAR. Based on the order of the epidemic degree, all the 692 townships were classified from high to low degrees. Among them, 127 townships had prevalence rates ≥ 1%. The high prevalence of human echinococcosis in TAR, which is associated with a wide geographic distribution, is a medical concern. Approximately 94.65% of the villages and towns reported echinococcosis cases. According to spatial distribution analysis, the prevalence of human echinococcosis was found to be clustered, with the specific clustering areas being identified. The cystic echinococcosis primary cluster covered 88 townships, while that of alveolar echinococcosis’s covered 38 townships.

**Conclusions:**

This study shows spatial distributions of echinococcosis with different epidemic degrees in 692 townships of TAR and high-risk cluster areas at the township level. Our findings indicate that strengthening the echinococcosis prevention and control strategies in TAR should directed at townships with a high prevalence and high-risk clustering areas.

**Graphical Abstract:**

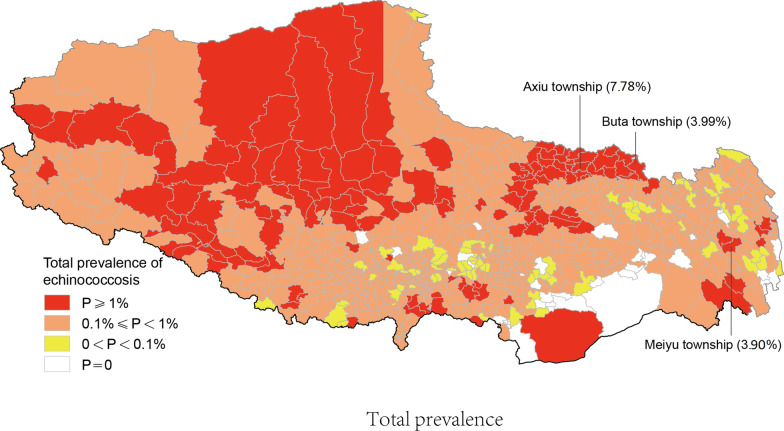

## Background

Echinococcosis, which is commonly known as hydatidosis, is a zoonotic parasitic disease caused by the larvae of *Echinococcus.* Globally, this disease is highly prevalent in pastoral areas of many countries [1]. In endemic areas of China, the number of patients with echinococcosis was estimated to be 166,098 in 2016 [2]. There are two major types of echinococcosis: cystic echinococcosis (CE) which is caused by the larvae of *E. granulosus* and alveolar echinococcosis (AE) which is caused by the larvae of *E. multilocularis* [3].

Echinococcosis is a long-course disease that mainly affects the liver, but also, the lungs and spleen [4, 5]. As the disease progresses, the infected organs deteriorate, causing organ dysfunction and eventually, death [4]. AE, also known as “parasitic cancer”, is the most lethal parasitic zoonosis with a 10-year mortality rate of more than 90% if untreated [4, 6]. Echinococcosis is a major public health concern with a substantive economic burden [3, 7, 8].

A national echinococcosis survey in China, conducted between 2012 and 2016, showed that 31 provincial-level administrative divisions (PLADs) had reported cases of echinococcosis. Within these PLADs, 370 counties reported local endemic human echinococcosis cases. These cases were mainly detected in the pastoral, semi-agricultural and semi-pastoral areas of TAR, Sichuan, Qinghai, Xinjiang, Gansu, Ningxia, Inner Mongolia, Yunnan and Shaanxi [2]. In addition, echinococcosis is closely associated with poverty among farmers and herdsmen [9]. In 2016, the prevalence of echinococcosis in TAR was 1.66%, with the estimated number of cases being 49,935 [2]. The average infection rates for intermediate hosts (cattle and sheep) as well as dogs were 13.21% and 7.30%, respectively [2]. These infections are highly associated with the prevalence of human echinococcosis [10]. In China, and globally, TAR is the region with the highest prevalence of echinococcosis [2]. In 2016, a sampling survey revealed that all the 74 counties under TAR jurisdiction were epidemic. All 692 townships in the 74 counties in 6 prefecture-level cities (Lhasa City, Changdu city, Shannan city, Shigatse city, Naqu city, Linzhi city) and one prefecture (Ali Prefecture) have been recognized as endemic areas for echinococcosis [2]. Given the severe epidemic situation, and the serious public health challenges associated with echinococcosis, a general census of human echinococcosis was performed in 2017. This census, which revealed an echinococcosis rate of 90.34%, was aimed at informing on suitable strategies for early detection and diagnosis. All echinococcosis patients were identified and, with their informed consents, treated with albendazole. Some of the patients were recommended for surgical treatment. In 2018, comprehensive measures for echinococcosis prevention and control were implemented in the 74 epidemic counties. However, implementation of these measures was challenging due to the complex natural environment, harsh climatic conditions, inadequate infrastructure, poor agricultural and animal husbandry practices, lagging social and economic development, delayed prevention and disease control strategies, difficult living and working conditions, outdated medical and health facilities as well as equipment, lack of vehicles suitable for the complex road conditions, sparsely populated areas, large service radius, and lack of professional as well as technical personnel [8, 10–13]. Since the launch of the National Echinococcosis Prevention and Control Project in 2008, the county level is the relevant level for defining the epidemic risk and allocation of health resources [14]. However, variations in epidemic degrees among townships within the same county have been found to significantly differ, with different epidemic characteristics. Therefore, scaling down the epidemic region from the county level to the township level is required to appropriately allocate health resources and improve the efficiencies of prevention and control measures. The risk of echinococcosis transmission has been comprehensively assessed. However, data for AE and CE elucidate on disease dynamics and on its relative distributions depending on the pathogen. CE and AE exhibit different transmission cycles and epidemic characteristics, and require different prevention and control measures. Therefore, careful analysis of relative distributions of CE and AE is necessary for designing relevant control measures that are suitable to local conditions, classify guidance, and improve efficiency.

Elucidation of the prevalence of echinococcosis at the township level in TAR is very important for prevention and control. In this study, we investigated the prevalence of the two main species of medical interest (AE and CE) in all 692 townships in TAR. Our findings will inform the development of suitable prevention and control measures and help in rational allocation of health resources.

## Methods

### Data source

Demographic data for each of the 692 townships of 74 endemic counties of TAR were obtained from the population survey released by the National Bureau of Statistics in 2018. Clinically diagnosed and confirmed cases of human echinococcosis were obtained from the annual report system of echinococcosis of the Tibet Center for Disease Control and Prevention. Most of the patients were diagnosed by echinococcosis census completed by the local health departments in their villages. Since echinococcosis has a long incubation period, the TAR census of echinococcosis was performed among people aged two and over in 692 townships. This census was complete by the end of 2018. Human echinococcosis cases were diagnosed by B-ultrasonography following the official “Diagnostic criteria for echinococcosis” of China (WS 257–2006), which is in line with that of World Health Organization [15]. Based on this strategy, the diagnostic criteria for CE by ultrasonography includes: a) Unilocular anechoic lesions that are round or oval with clearly visible cyst walls (laminated layers) and snowflake-like inclusions or floating laminated membranes; b) Multivesicular or multiseptate cysts with wheel-like appearances; and c) Unilocular cysts with daughter cysts and honeycomb appearances. The diagnostic criteria for AE include: the presence of lesions that are characterized by heterogenous hypodense masses, often associated with necrotic cavities, irregular lesion contours and lack of well-defined walls [15]. Serological tests were performed for patients exhibiting space occupying lesions and live or work in epidemic areas or for those reporting contact histories with domestic or wild animals and their fur. These tests were performed by enzyme-linked immunosorbent assay (ELISA) (Zhuhai Hai Tai Biopharmaceutical Co., Ltd. Zhuhai, China). Suspected cases were confirmed by positive serological tests [15]. After obtaining their informed consents, the identified patients were treated with albendazole and recommended for surgical treatment [15]. Data were analyzed at the township level.

### Classification of prevalence

Based on official “Diagnostic criteria for echinococcosis” of China (WS 257–2006), echinococcosis cases were classified into CE, AE, and co-infections with CE and AE [15]. The prevalence of CE was determined by including the confirmed cases of CE as well as the co-infections of CE with AE in the calculations. Similarly, the prevalence of AE was obtained by including the confirmed cases of AE as well as co-infections of CE with AE in the calculation. Classification of the prevalence of human echinococcosis was based on classification standards for endemic counties as reported in the 2019 edition of technical guidelines for echinococcosis control in China [16]. Due to differences in transmission cycles, preventive strategies, control measures, clinical manifestations, as well as treatment regimens, the characteristics of CE and AE were independently described and analyzed. Each case was classified based on epidemic status, from the highest to the lowest, to provide a qualitative understanding of the epidemic status of echinococcosis in given townships. The 2019 edition of technical guidelines for echinococcosis control in China recommend that evaluation of echinococcosis control and elimination measures should be based at the township level [16]. Since the current epidemic areas of echinococcosis in China were only considered at the county level and no classification standards for township level are available, we classified the epidemic status of echinococcosis in all townships in TAR following the relevant standards of population prevalence index in the national technical plan for echinococcosis control (2019 Edition). The classification criteria were: Class I epidemic townships: prevalence rate ≥ 1%; Class II epidemic townships: prevalence rate ≥ 0.1% and < 1%; Class III epidemic townships: prevalence rate ≥ 0 and < 0.1%; Class IV epidemic townships: townships with appropriate transmission circulation conditions and a prevalence rate equal to 0 [16]. Statistical analyses was performed using the SPSS 21.0 software package (IBM, Armonk, USA). The ArcGIS 10.1 program (ESRI, Redlands, USA) was used for geographic mapping and prevalence analysis.

### Spatial scan clustering

SaTScan V9.5 (Management Information Services, Maryland, USA) was used for retrospective spatial scan analysis. The spatial clustering scanning analysis was based on echinococcosis cases and exposed population in the 692 townships by the end of 2018. The discrete Poisson probability model was applied using a circular window for high-rate clusters. Areas of high incidence were scanned using a moving circular window, dynamically varying in size. The maximum sizes of spatial and temporal windows were defined as 25% of the total population of the entire area. Likelihood ratio tests and Monte Carlo randomization tests were used to determine the significance of spatiotemporal clusters. Finally, the window with the maximum Log-likelihood ratio (*LLR*) value was defined as the primary cluster while other clusters with statistically significant *LLR*s were defined as secondary clusters and minor secondary clusters. The radius of minor secondary clusters was less than 50 km. Relative risk (*RR*) and *P*-value for each cluster were obtained by Monte Carlo randomization tests. W randomization data sets were generated by Monte Carlo randomization tests. The maximum *LLR* were calculated in the same way as the observed data and were sorted from large to small. If the maximum *LLR* of the real data set is ranked R, then *P* = R/(W + 1). The higher the ranking, the smaller the *P*-value, indicating that the probability for random aggregation is smaller. Scan results were visualized using the Arcgis10.1 software (ESRI, Redlands, USA).

## Results

### Prevalence of human echinococcosis

From a total of 3,002,828 people, there were 16,009 (0.53%) echinococcosis-positive patients. In 2018, the total population of Tibet was 3,324,078 with the census rate of echinococcosis being 90.34% [17]. Among them, there were 14,398 (89.94%) CE cases, 942 (5.88%) AE cases and 137 (0.86%) co-infections of CE and AE. The proportions of male and female cases were 35.4% and 64.6%, respectively. The youngest in age was 2 years old, while the oldest was 93 years with a median age of 46 years. Due to a lack of detailed classification records and failure to classify them as cystic or alveolar echinococcosis, a total of 532 cases (3.32%) were unclassified (Table 1).

**Table 1:** Epidemic status of human echinococcosis in Tibet Autonomous Region, 2018

Prefecture/Prefecture-level (municipal level) city	Number of endemic counties	Population of endemic areas	All cases					Prevalence rate (1/10,000)	Prevalence rate of CE (1/10,000)	Prevalence rate of AE (1/10,000)
			Total cases	CE	AE	Co-infection of CE and AE cases	Unclassified cases			
Lhasa	8	477,334	958	932	3	1	22	20.07	19.55	0.08
Changdu	11	723,005	2856	2266	312	54	224	39.50	32.09	5.06
Shannan	12	306,813	1295	1259	1	7	28	42.21	41.26	0.26
Shigatse	18	772,334	3147	3025	10	12	90	40.75	39.32	0.28
Naqu	11	478,172	6019	5273	603	44	99	125.88	111.19	13.53
Ali	7	103,155	1075	986	3	17	69	104.21	97.23	1.94
Linzhi	7	141,995	659	647	10	2	0	46.41	45.71	0.85
Total	74	3,002,828	16,009	14,398	942	137	532	53.31	48.40	3.59

*AE* Alveolar echinococcosis, *CE* Cystic echinococcosis

**Fig. 1:**
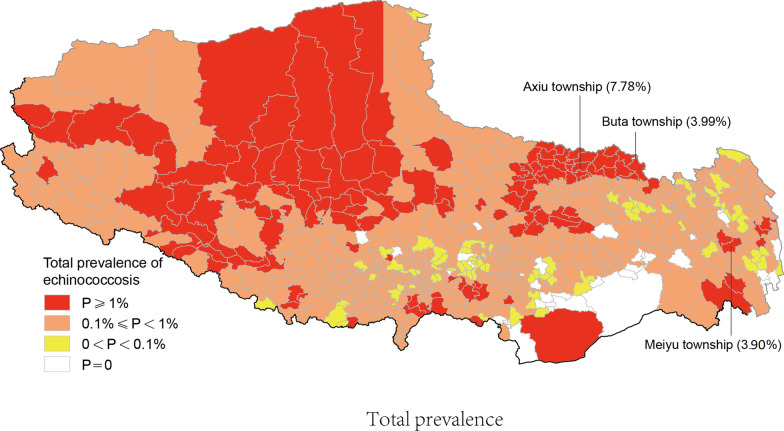
Spatial distribution of human echinococcosis. P: Prevalence

The prevalence of human echinococcosis at the township level ranged from 0 to 7.78% in TAR. The three townships with the highest prevalence rates were Axiu township in Baqeen County of Naqu Prefecture-level city with 216/2775 (7.78%) cases, Buta township in Deengqeen County with 130/3260 (3.99%) cases, and Meiyu township in Zogang county of Changdu Prefecture-level city with 201/5152 (3.90%) cases. All cases were distributed in 655 townships. However, there were no cases of echinococcosis in 37 townships (Fig. 1, Table 2).

**Table 2:** Classification of the prevalence of human echinococcosis at township level in Tibet Autonomous Region, 2018

District/ Prefecture-level (municipal level) city	Total number of towns	P ≥ 100/10,000 (Class I epidemic townships)		10/10,000 ≤ P < 100/10,000 (Class II epidemic townships)		0 < P < 10/10,000 (Class III epidemic townships)		P = 0 (Class IV epidemic townships)	
		Number of towns	Constituent ratio (%)	Number of towns	Constituent ratio (%)	Number of towns	Constituent ratio (%)	Number of towns	Constituent ratio (%)
Lhasa	65	1	1.54	39	60.00	22	33.85	3	4.62
Changdu	138	11	7.97	95	68.84	24	17.39	8	5.80
Shannan	82	8	9.76	46	56.10	14	17.07	14	17.07
Shigatse	203	24	11.82	157	77.34	19	9.36	3	1.48
Naqu	114	66	57.89	46	40.35	2	1.75	0	0.00
Ali	37	15	40.54	22	59.46	0	0.00	0	0.00
Linzhi	53	2	3.77	41	77.36	1	1.89	9	16.98
Total	692	127	18.35	446	64.45	82	11.85	37	5.35

*P* Prevalence, *AE* Alveolar echinococcosis, *CE* Cystic echinococcosis

**Fig. 2:**
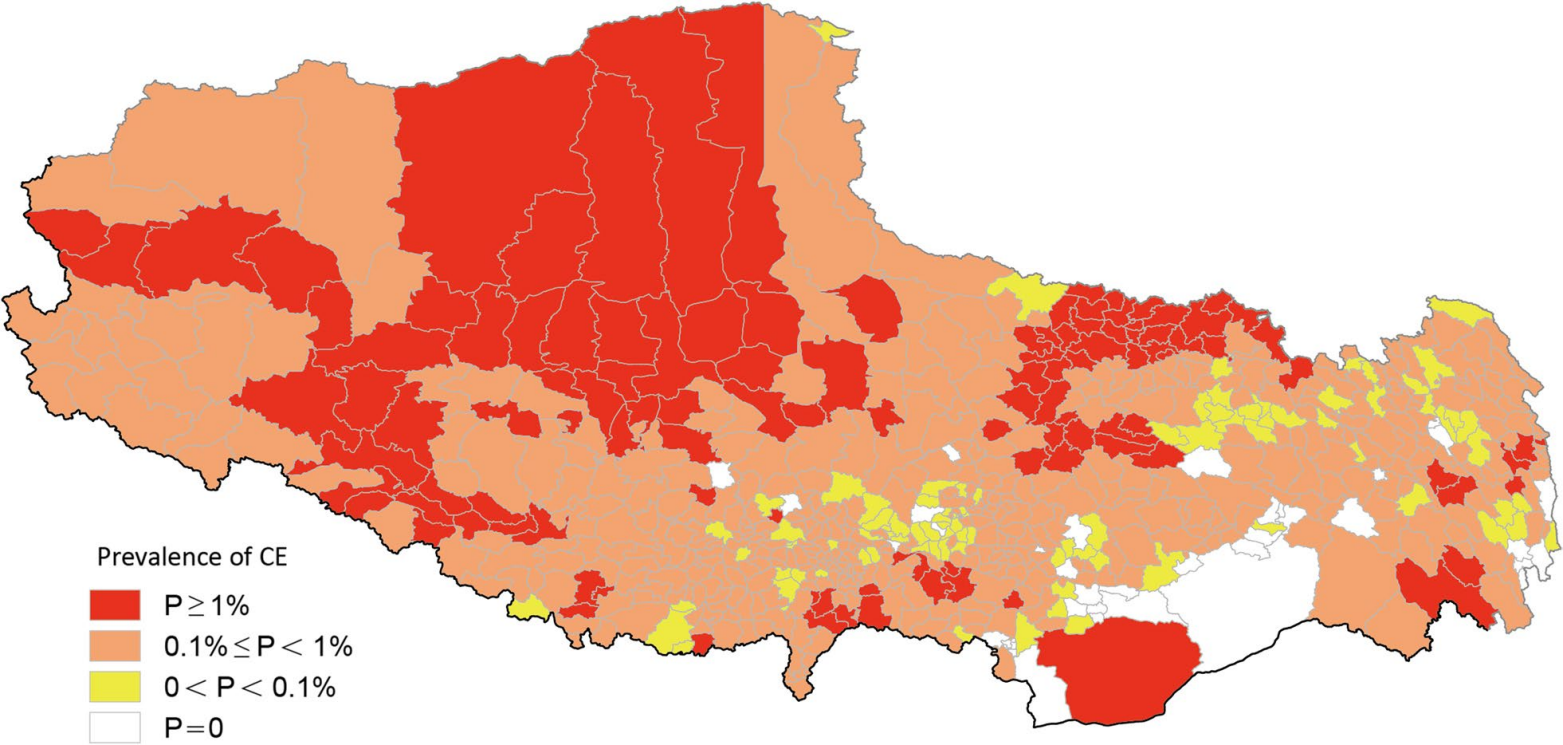
Spatial distribution of human CE. P: Prevalence

**Fig. 3:**
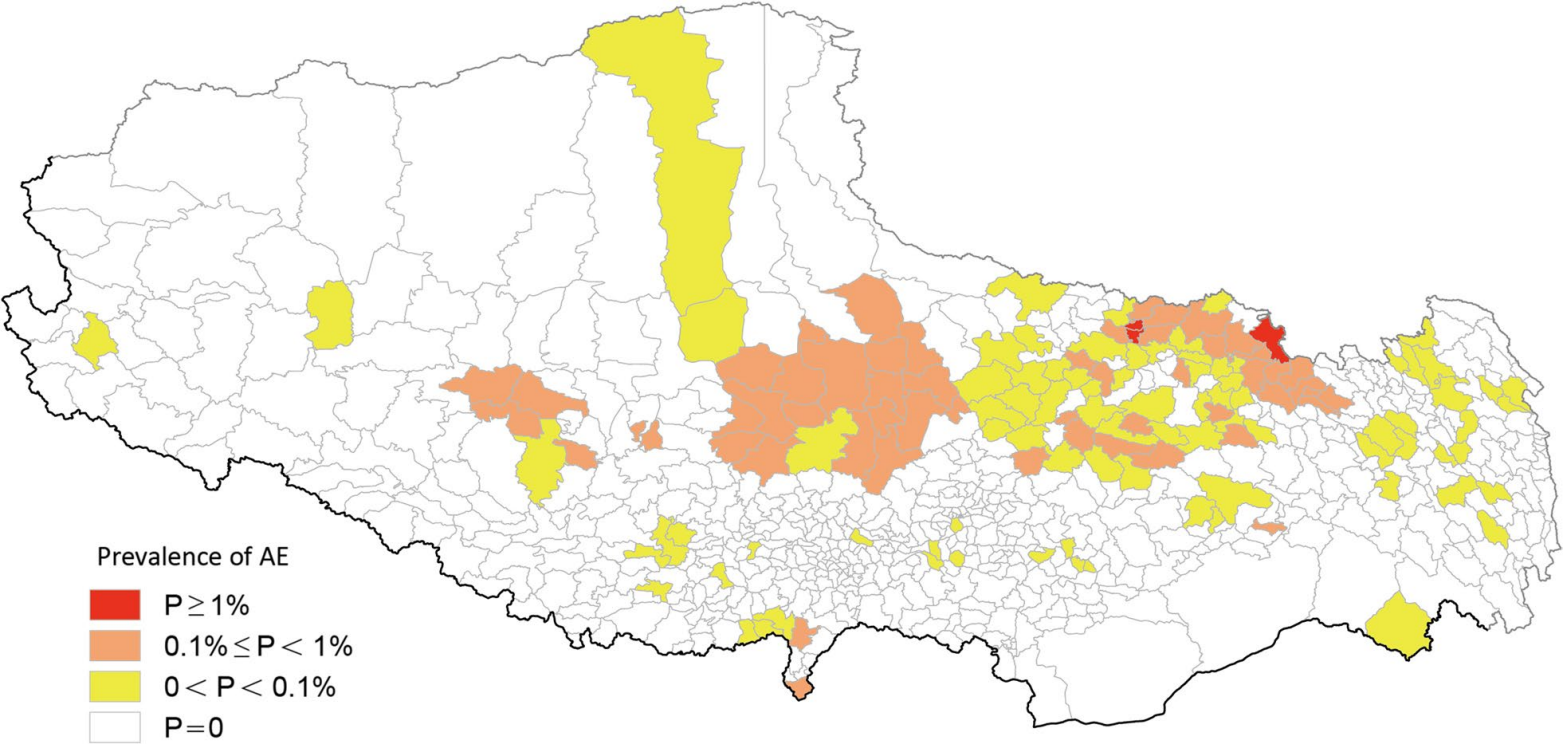
Spatial distribution of human AE. AE: Alveolar echinococcosis, P: Prevalence

The overall prevalence rate of CE was 0.48%, and all cases were distributed in 655 townships of 74 counties. The three townships with the highest prevalence of CE were Axiu township in Baqeen County of Naqu Prefecture-level city with 171/2775 (6.16%) cases, Meiyu township in Zogang County of Changdu Prefecture-level city with 201/5152 (3.90%) cases, and Baixiong township in Nierong County of Naqu Prefecture-level city with 144/4325 (3.33%) cases (Fig. 2, Table 3). The overall prevalence of AE was 0.04%, with the 1079 recorded cases being found to be distributed in 143 townships from 32 counties. The three townships with the highest prevalence for AE were Axiu township with 46/2775 (1.66%) cases, Baqeen township with 35/2300 (1.52%) cases, both of which are in Baqeen County of Naqu Prefecture-level city, and Buta township in Deengqeen County with 34/3260 (1.04%) cases (Fig. 3, Table 4).

**Table 3:** Classification of the prevalence of human cystic echinococcosis at township level in Tibet Autonomous Region, 2018

District/ Prefecture-level (municipal level) city	Total number of towns	P ≥ 100/10,000 (Class I epidemic townships)		10/10,000 ≤ P < 100/10,000 (Class II epidemic townships)		0 < P < 10/10,000 (Class III epidemic townships)		P = 0 (Class IV epidemic townships)	
		Number of towns	Constituent ratio (%)	Number of towns	Constituent ratio (%)	Number of towns	Constituent ratio (%)	Number of towns	Constituent ratio (%)
Lhasa	65	1	1.54	37	56.92	24	36.92	3	4.62
Changdu	138	9	6.52	90	65.22	31	22.46	8	5.80
Shannan	82	7	8.54	46	56.10	15	18.29	14	17.07
Shigatse	203	24	11.82	158	77.83	18	8.87	3	1.48
Naqu	114	61	53.51	49	42.98	4	3.51	0	0.00
Ali	37	12	32.43	25	67.57	0	0.00	0	0.00
Linzhi	53	2	3.77	40	75.47	2	3.77	9	16.98
Total	692	116	16.76	445	64.31	94	13.58	37	5.35

*P* Prevalence, *AE* Alveolar echinococcosis, *CE* Cystic echinococcosis

**Table 4:** Classification of the prevalence of human alveolar echinococcosis at township level in TAR, 2018

District/ Prefecture-level (municipal level) city	Total number of towns	P ≥ 100/10,000 (Class I epidemic townships)		10/10,000 ≤ P < 100/10,000 (Class II epidemic townships)		0 < P < 10/10,000 (Class III epidemic townships)		P = 0 (Class IV epidemic townships)	
		Number of towns	Constituent ratio (%)	Number of towns	Constituent ratio (%)	Number of towns	Constituent ratio (%)	Number of towns	Constituent ratio (%)
Lhasa	65	0	0.00	0	0.00	2	3.08	63	96.92
Changdu	138	1	0.72	12	8.70	26	18.84	99	71.74
Shannan	82	0	0.00	0	0.00	6	7.32	76	92.68
Shigatse	203	0	0.00	4	1.97	10	4.93	189	93.10
Naqu	114	2	1.75	33	28.95	33	28.95	46	40.35
Ali	37	0	0.00	3	8.11	3	8.11	31	83.78
Linzhi	53	0	0.00	1	1.89	7	13.21	45	84.91
Total	692	3	0.43	53	7.66	87	12.57	549	79.34

*P* Prevalence, *AE* Alveolar echinococcosis, *CE* Cystic echinococcosis

### Classification of the prevalence of human echinococcosis in China

The epidemic levels of 692 townships in TAR were: 127 (18.35%) were Class I epidemic townships; 446 (64.45%) were Class II epidemic townships; 82 (11.85%) were Class III epidemic townships; while 37 (5.35%) were Class IV epidemic townships (Fig. 1, Table 2). The classifications of CE and AE were further analyzed based on similar classification criteria. Among the 692 townships in TAR, 655 (94.55%) had CE cases with 116 (16.76%) of them being Class I epidemic townships, 445 (64.31%) being Class II epidemic townships, 94 (13.58%) being Class III epidemic townships and 37 (5.35%) being Class IV epidemic townships (Fig. 2, Table 3). With respect to AE, 143 (20.7%) out of 692 townships had AE cases with 3 (0.43%) of them being Class I epidemic townships, 53 (7.66%) being Class II epidemic townships, 87 (12.57%) being Class III epidemic townships and 549 (79.34%) being Class IV epidemic townships (Fig. 3, Table 4).

### Spatial distribution and identification of clusters of human echinococcosis

#### Spatial clustering of human echinococcosis

**Fig. 4:**
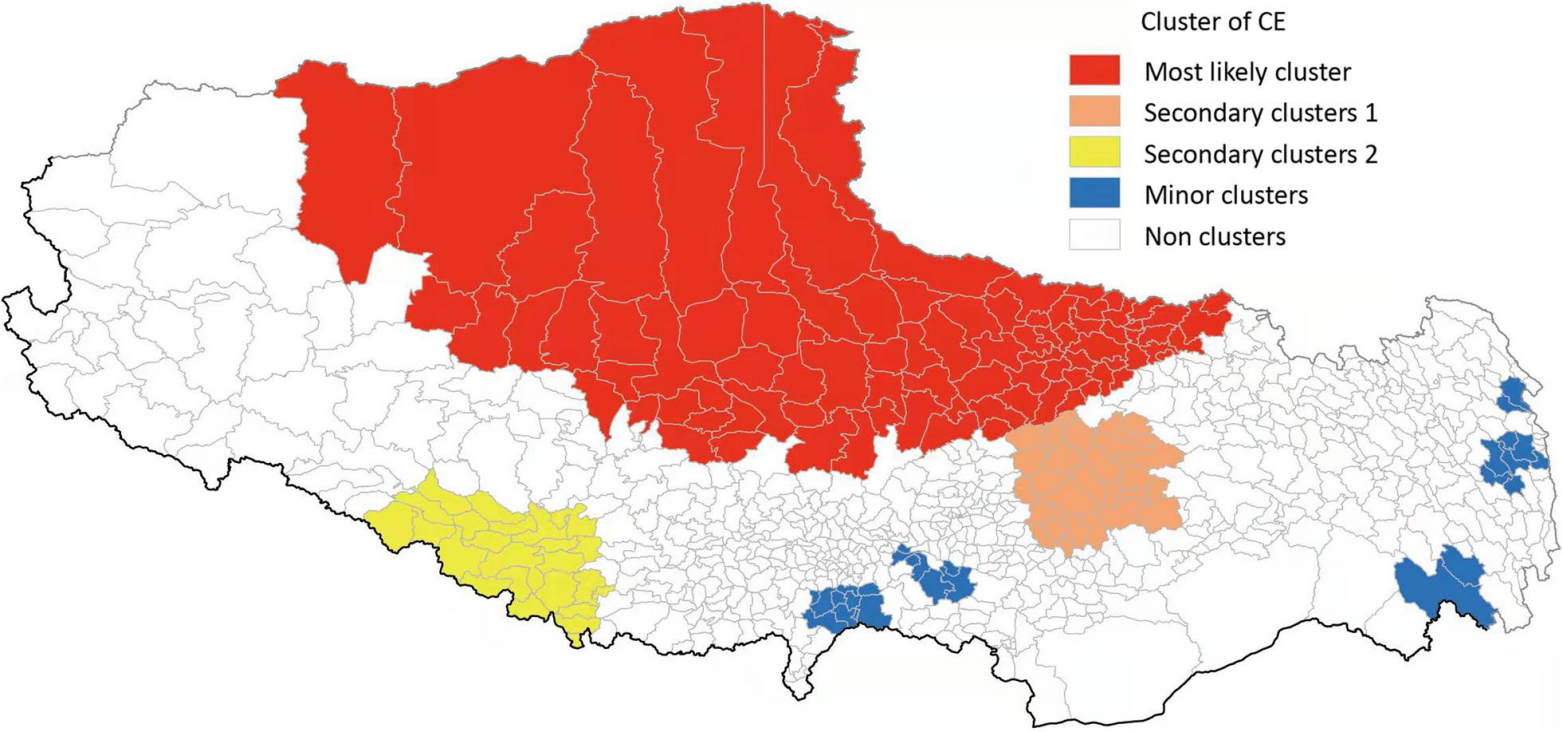
SaTScan spatial clustering analysis of human CE. CE: Cystic echinococcosis

The analysis of CE revealed one primary cluster and seven secondary clusters. The primary cluster was centered at 36°10’ North and 89°39’ East with a radius of 632.91 km, covering 88 townships in 12 counties. It was dominated by the Naqu Prefecture-level city, with 82 townships in all the 10 epidemic counties (Nagqum, BIrum, Nyainrong, Amdo, Xainza, Sog, Bangoin, Baqeen, Nyima, and Shuanghu), followed by Damxung in Lhasa city and Geerzee in the Ali Prefecture. This cluster had 356,976 exposed persons, with a risk of infection 3.35 times higher than in other areas (*P* < 0.01). It is the key area in TAR for the prevention and control of CE. The extent and risk status of secondary clusters are shown in Fig. 4 and Table 5. A relatively important secondary cluster area was centered at 30°11’ North and 92°86’ East with a radius of 103.61 km, covering 27 towns from 7 counties, including Maizhokunggar in Lhasa city, Sangri and Gyaca of Shannan Prefecture-level city, Nagqu and Jiali in Naqu Prefecture-level city, as well as Nyingchi and Gongbo’gyamda in Linzhi Prefecture-level city. The *RR* value of this cluster was 1.77 (*P* < 0.01). Another important secondary cluster area was centered at 28°75’ North and 84°83’ East with a radius of 151.51 km, covering 25 towns from 6 counties. The *RR* value of this cluster was 1.71 (*P* < 0.01). The remaining secondary clusters were relatively small, involving only a few townships.

**Table 5:** Spatial clustering analysis of human cystic echinococcosis in Tibet Autonomous Region, 2018

Cluster	Center point		Scope		Radius(km)	Exposed population	Number of cases	Number of expected cases	*RR*	*LLR*	*P*-value
	Latitude	Longitude	Center town	Number of towns							
Primary cluster	36.099499 N	89.386002 E	Sewu town of Amdo county	88	632.91	356,976	4494	1716	3.35	1,876.79	< 0.01
Secondary cluster1	30.110399 N	92.856300 E	Jinda town of Gongbo' gyamda county	27	103.61	98,424	815	473	1.77	105.65	< 0.01
Secondary cluster2	28.750000 N	84.828003 E	Gongdang town of Gyirong county	25	151.51	53,153	431	255	1.71	51.00	< 0.01
Minor secondary cluster1	28.343500 N	89.611000 E	Samada town of Kangmar county	9	48.71	18,832	219	91	2.44	65.61	< 0.01
Minor secondary cluster2	30.401300 N	98.491096 E	Latuo town of Konjo county	7	37.23	22,215	233	107	2.20	56.17	< 0.01
Minor secondary cluster3	28.755199 N	91.116699 E	Gongbuxue town of Nagarzee county	3	30.80	16,538	371	79	4.76	283.07	< 0.01
Minor secondary cluster4	29.071199 N	90.505997 E	Kalong town of Nagarzee county	4	20.62	8,920	98	43	2.29	26.01	< 0.01
Minor secondary cluster5	28.648899 N	97.541801 E	Zhuwagen town of Zayuu county	2	47.02	6,316	73	30	2.41	21.48	< 0.01
Minor secondary cluster6	31.132999 N	98.431099 E	Niangxi town of Jomda county	2	21.23	10,820	104	58	1.80	14.96	< 0.05

*LLR* Log-likelihood ratio, *RR* Relative risk

**Fig. 5:**
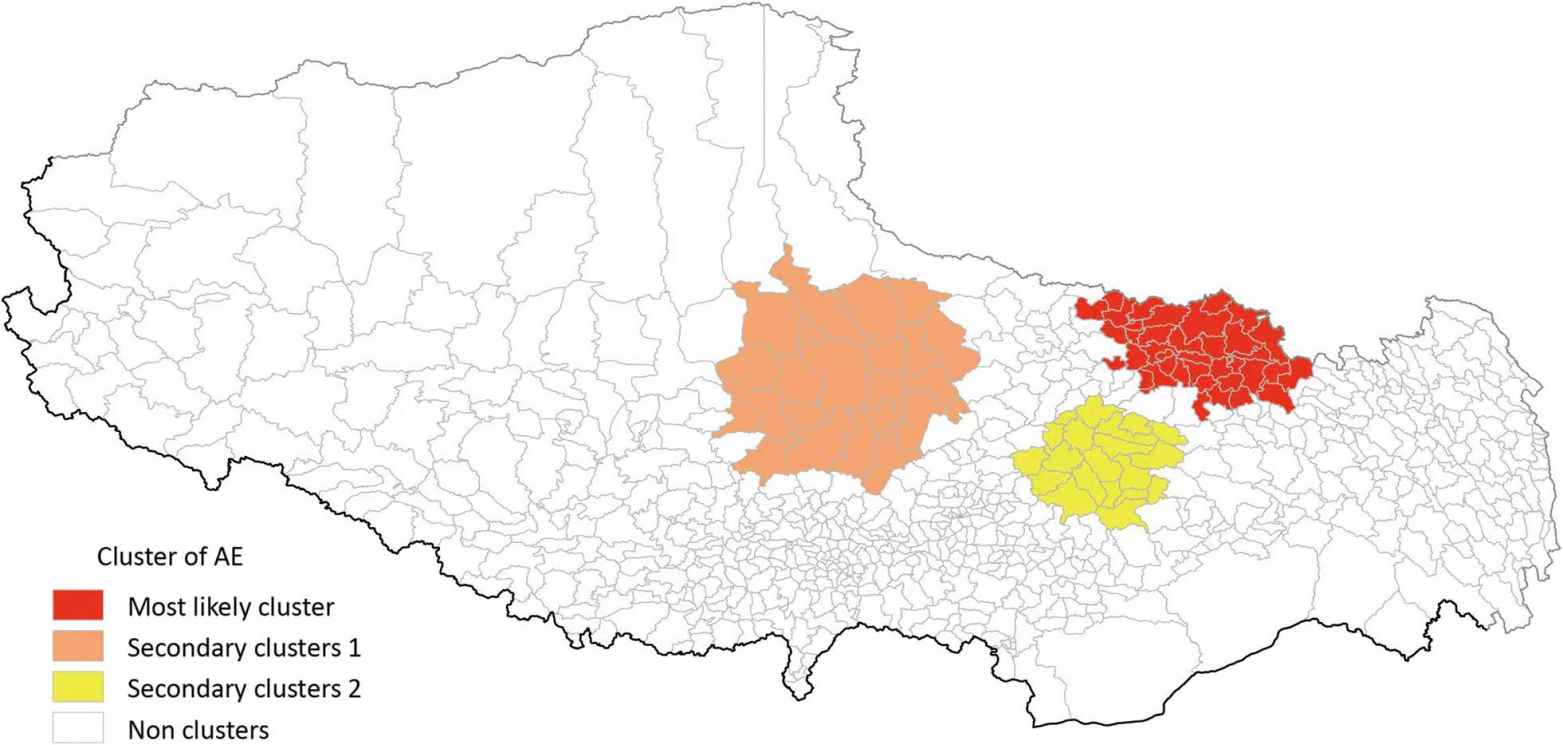
SaTScan spatial clustering analysis of human AE. AE: Alveolar echinococcosis

Spatial clustering scans of AE revealed the presence of one primary cluster and two secondary clusters (Fig. 5 and Tables 6 and 7). The primary cluster was centered at 32°49’ North and 94°54’ East, with Gongri township in Baqeen county being the center. The radius of this cluster was 157.23 km, covering 38 townships in 6 counties, including Deengqeen and Banbar in Changdu Prefecture-level city as well as Biru, Nyainrong, Sog, and Baqeen in Naqu Prefecture-level city. The *RR* value of this cluster area was as high as 21.04 times that of the surrounding area (*P* < 0.01).

**Table 6:** Spatial clustering analysis of human alveolar echinococcosis in Tibet Autonomous Region, 2018

Cluster	Center point		Scope		Radius(km)	Exposed population	Number of cases	Number of expected cases	*RR*	*LLR*	*P*-value
	Latitude	Longitude	center town	Number of towns							
Primary cluster	32.494598 N	94.544701 E	Gongri town of Baqeen county	38	157.23	194,212	557	61	21.04	916.09	< 0.01
Secondary cluster1	31.559900 N	89.523499 E	Mendang town of Bangoin county	22	158.35	70,604	152	22	8.02	173.05	< 0.01
Secondary cluster2	30.342400 N	93.036400 E	Niangpu town of Gongbo' gyamda county	20	93.93	69,309	54	22	2.58	17.56	< 0.01

*LLR* Log-likelihood ratio, *RR* Relative risk

**Table 7:** The correspondence table between the names of 74 counties involved in this paper and the names of Chinese Pinyin

Prefecture/Prefecture-level city (municipal level)	County name involved in the article	County name in Chinese pinyin	Prefecture/Prefecture-level city (municipal level)	County name involved in the article	County name in Chinese pinyin
Lhasa	Chengguan	Chengguan		Ngamring	Angren
	Lhuunzhub	Linzhou		Xaitongmoin	Xietongmen
	Damxung	Dangxiong		Bainang	Bailang
	Nyeemo	Nimu		Rinbung	Renbu
	Quuxuu	Qushui		Kangmar	Kangma
	Doilungdeeqeen	Duilong Deqing		Dinggyee	Dingjie
	Dagzee	Dazi		Zhongba	Zhongba
	Maizhokunggar	Mozhu Gongka		Yadong(Chomo)	Yadong
Changdu	Karuo	Karuo		Gyirong	Jilong
	Jomda	Jiangda		Nyalam	Nielamu
	Konjo	Gongjue		Saga	Saga
	Riwoqee	Leiwuqi		Gamba	Gangba
	Deengqeen	Dingqing	Naqu	Nagqu	Naqu County
	Chagyab	Chaya		Jiali(Lhari)	Jiali
	Baxoi	Basu		Biru	Biru
	Zogang	Zuogong		Nyainrong	Nierong
	Mangkam	Mangkang		Amdo	Anduo
	Lhorong	Luolong		Xainza	Shenza
	Banbar	Bianba		Sog	Suoxian
Shannan	Needong	Naidong		Bangoin	Bange
	Chanang	Zanang		Baqeen	Baqing
	Gonggar	Gongga		Nyima	Nima
	Sangri	Sangri		Shuanghu	Shuanghu
	Qonggyai	Qiongjie	Ali	Burang	Pulan
	Qusum	Qusong		Zanda	Zhada
	Comai	Cuomei		Gar	Gaer
	Lhozhag	Luoza ^b^		Rutog	Ritu
	Gyaca	Jiacha		Gee'gyai	Geji
	Lhuunzee	Longzi		Geerzee	Gaize
	Cona	Cuona		Coqeen	Cuoqin
	Nagarzee	Langkazi	Linzhi	Nyingchi	Linzhi County
Shigatse	Xigazee	Sangzhuzi		Gongbo'gyamda	Gongbu Jiangda
	Namling	Nanmulin		Mainling	Milin
	Gyangzee	Jiangzi		Metog	Motuo
	Tingri	Dingri		Bomi(Bowo)	Bomi
	Sa'gya	Sajia		Zayuu	Chayu
	Lhazee	Lazi		Nang	Langxian

One secondary cluster was centered at 31°56’ North and 89°52’ East, with Mendang Township in Bangoin County being at the center. This cluster had a radius of 158.35 km, covering 22 townships in 4 counties in the Naqu Prefecture-level city, including 10 townships in Bangoin County, 7 townships in Xainza County, 1 township in Suanghu County and 1 in Amdo County. The *RR* value of this cluster area was 8.02. The risk of AE transmission in this aggregation area was significantly higher than that of the surrounding area.

With Niangpu Township in Gongbo' gyamda county as the center, the other secondary cluster was centered at 30°34’ North and 93°04’ East. It had a radius of 93.93 km, covering 20 townships in 4 counties, including 8 townships in Gongbo' gyamda county, 10 townships in Jiali county, 1 township in MaizhoKunggar county and 1 in Banbar county. The *RR* value of this cluster area was 2.58 (*P* < 0.01).

## Discussion

Both primary clusters for CE and AE, which had the largest number of Class I epidemic townships, covered the townships in Naqu prefecture-level city. These townships are located in the northern Tibet Plateau, whose average altitude is more than 4500 m and is a major pastoral area of Tibet. The Naqu Prefecture-level city of TAR has the highest prevalence of both AE and CE. As a pastoral area, there are many dogs, livestock, and wild animals. Particularly, the high prevalence of AE is associated with the high abundance of stray dogs [18, 19]. These factors form the transmission cycles of CE and AE. Moreover, local residents have poor health habits and living conditions, including unclean domestic water due to poor natural environments and backward economic development. Local medical conditions are also limited. In most parts of TAR, like in the Naqu Prefecture-level city, local residents depend on pastoral work for living, with same traditional production and life styles, poor medical and health resources as well as shallow health awareness, which predisposes them to echinococcosis. Besides, knowledge on disease and appropriate prevention methods is generally low [2, 20].

Our findings show that compared to CE, the prevalence of AE in TAR is relatively low, and its distribution is relatively limited, in tandem with findings from other studies [2]. The transmission cycle of AE involves sylvatic cycles, with foxes and dogs as definitive hosts and small rodents as intermediate hosts. The distribution of small rodents is less than that of livestock. However, given the heavy disease burden on AE patients, the 56 AE epidemic class I and class II townships and the three AE cluster areas should be prioritized. In addition to strengthening the control of infection at source levels and health education measures, it is important to increase the surveillance of small rodents and lagomorphs (alternative intermediate hosts of *E*. *multilocularis*), strictly control the number of stray dogs, and strengthen the screening of AE patients in these townships [24]. In China, the primary definitive host for both *E*. *granulosus* and *E*. *multilocularis* is dog [21, 22]. Therefore, monitoring the number of dogs and regular deworming of infected dogs is an important prevention and control approach [23]. In class I and II epidemic townships, each dog must be dewormed monthly, consistent with the required prevention and control measures. In addition, health education and people awareness should be intensified. In townships with insufficient health workers, rural cadres and volunteers should be actively recruited and trained to promote the implementation of this measure. In class III townships, the deworming frequency can be rationally reduced according to the actual situation. Deworming of dogs during the slaughtering season of intermediate hosts, such as yaks and sheep, as well as the strengthening of health education, awareness and slaughter management procedures must be implemented. Moreover, the intensification of health education and awareness for residents, as well as echinococcosis monitoring should be performed in class IV epidemic townships.

The relevant departments of epidemic counties in which these towns belong should pay more attention to the epidemic towns in the gathering areas, and strengthen the monitoring of echinococcosis and patient screening. If the epidemic areas involve the junction areas of multiple epidemic counties, the relevant epidemic counties should cooperate and implement the relevant prevention and control strategies. The spatial distribution of AE is more limited, with only three aggregation areas, however, the epidemic risk is relatively high, especially in the primary cluster area, suggesting that these aggregation areas require strengthening of prevention and control measures. The populations should be actively mobilized to participate in echinococcosis prevention and control strategies. Implementation of prevention and control approaches that are well adapted to the local reality and with high economic effects should be prioritized to significantly reduce disease burden and efficiently control echinococcosis epidemics. There were 532 unclassified cases in 93 townships, and in some townships it was true for all cases. This might seriously affect the management and prevention of echinococcosis; therefore, this issue should be addressed as a priority. B-mode ultrasound technicians should be trained at the township level. Quality control and supervision of the integrity of patient records and materials should also be improved. Based on our findings, when allocating health resources, the relevant administrative departments should focus on townships with a high prevalence and primary cluster areas.

This study is associated with some limitations. First, as a chronic infectious parasitic disease, echinococcosis is characterized by occult onsets and long incubation periods. Therefore, although the spatial distribution of prevalence can reflect the disease burden and historical risk of echinococcosis in different areas, it cannot sensitively reflect the current infection risks. Second, populations were screened for hydatid lesions using portable B-mode ultrasonography, therefore, only abdominal lesions of CE and AE could be detected, whereas lesions in the lungs, brain, and other organs outside the abdomen could not be detected. Furthermore, most patients were identified by screening or clinical examination, but a part of the infected population was not identified. Some patients were leaked during detections, thus, the prevalence determined in the survey may be underestimated. Finally, for the 532 (3.3%) confirmed patients, there were no clear classifications (CE or AE) and neither were there clear B-mode ultrasound images, which may lead to failures in identification and treatment. This may affect the analysis, follow-up and treatment of patients.

## Conclusions

This study shows spatial distributions of echinococcosis with different epidemic degrees in 692 townships of TAR and high-risk cluster areas at the township level. There have been advances in the prevention and control of echinococcosis in TAR. Our findings provide a scientific reference for the relevant administrative departments in TAR to appropriately adjust the prevention and control strategies according to the different epidemic characteristics and epidemic degrees. Therefore, we suggest the formulation of different prevention and control measures in primary and secondary clusters. Future studies should formulate more advanced prevention and control strategies to efficiently prevent and control echinococcosis in TAR.

## Data Availability

All data analyzed in the present study are included in the article material. Any inquiries can be directed to the corresponding author.

## Abbreviations

AE: Alveolar echinococcosis
CE: Cystic echinococcosis
TAR: Tibet Autonomous Region
*LLR*: Log-likelihood ratio
*RR*: Relative risk

## References

[1] Vuitton DA, McManus DP, Rogan MT, Romig T, Gottstein B, Naidich A (2020). International consensus on terminology to be used in the field of echinococcoses. Parasite.

[2] Wu WP, Wang H, Wang Q, Zhou XN, Wang LY, Zheng CJ (2018). A nationwide sampling survey on echinococcosis in China during 2012–2016. Zhongguo Ji Sheng Chong Xue Yu Ji Sheng Chong Bing Za Zhi.

[3] Budke CM, Deplazes P, Torgerson PR (2006). Global socioeconomic impact of cystic echinococcosis. Emerg Infect Dis.

[4] McManus DP, Gray DJ, Zhang W, Yang Y (2012). Diagnosis, treatment, and management of echinococcosis. BMJ.

[5] Wen H, Vuitton L, Tuxun T, Li J, Vuitton DA, Zhang W (2019). Echinococcosis: advances in the 21st Century. Clin Microbiol Rev.

[6] Qucuo N, Wu G, He R, Quzhen D, Zhuoga C, Deji S (2020). Knowledge, attitudes and practices regarding echinococcosis in Xizang Autonomous Region, China. BMC Public Health.

[7] Cadavid Restrepo AM, Yang YR, McManus DP, Gray DJ, Giraudoux P, Barnes TS (2016). The landscape epidemiology of echinococcoses. Infect Dis Poverty.

[8] Fu MH, Wang X, Han S, Guan YY, Bergquist R, Wu WP (2021). Advances in research on echinococcoses epidemiology in China. Acta Trop.

[9] Wang Q, Francis R, Christine B, Philip SC, Xiao YF, Dominique A (2010). Grass height and 525 transmission ecology of *Echinococcus multilocularis* in Tibetan communities. Chin Med J (Engl).

[10] Gong QL, Ge GY, Wang Q, Tian T, Liu F, Diao NC (2021). Meta-analysis of the prevalence of *Echinococcus* in dogs in China from 2010 to 2019. PLoS Negl Trop Dis.

[11] Ito A, Urbani C, Jiamin Q, Vuitton DA, Qiu DC, Heath DD (2003). Control of echinococcosis and cysticercosis: a public health challenge to international cooperation in China. Acta Trop.

[12] Ertabaklar H, Dayanır Y, Ertuğ S (2012). Research to investigate human cystic echinococcosis with ultrasound and serologic methods and educational studies in different provinces of Aydın/Turkey. Turkiye Parazitol Derg.

[13] Chahed MK, Bellali H, Touinsi H, Cherif R, Ben SZ, Essoussi M (2010). Distribution of surgical hydatidosis in Tunisia, results of 2001–2005 study and trends between 1977 and 2005. Arch Inst Pasteur Tunis.

[14] Zhang WB, Zhang ZZ, Wu WP, Shi BX, Li J, Zhou XN (2015). Epidemiology and control of echinococcosis in central Asia, with particular reference to the People's Republic of China. Acta Trop.

[15] Ministry of Health of the People’s Republic of China. Diagnostic criteria of echinococcosis. Trop Dis Parasitol. 2018;01:56–61 (in Chinese).

[16] National Health Commission of the People’s Republic of China. Notice about 429 printing of echinococcosis prevention technology solutions (2019). http://www.gov.cn/zhengce/zhengceku/2020-09/03/content_5539743.htm. Accessed 30 Dec 2022.

[17] National Health Commission of the People’s Republic of China (2013). China Statistical Yearbook.

[18] Wang Q, Raoul F, Budke C, Craig PS, Xiao YF, Vuitton DA (2010). Grass height and transmission ecology of *Echinococcus multilocularis* in Tibetan communities. China Chin Med J (Engl).

[19] Giraudoux P, Pleydell D, Raoul F, Quere JP, Wang Q, Yang YR (2006). Transmission ecology of *Echinococcus multilocularis*: what are the ranges of parasite stability among various host communities in China. Parasitol Int.

[20] Li B, Quzhen G, Xue CZ, Han S, Chen WQ, Yan XL (2019). Epidemiological survey of echinococcosis in Tibet Autonomous Region of China. Infect Dis Poverty.

[21] Craig PS, Hegglin D, Lightowlers MW, Torgerson PR, Wang Q (2017). Echinococcosis: control and prevention. Adv Parasitol.

[22] Cai QG, Han XM, Yang YH, Zhang XY, Ma LQ, Karanis P (2018). Lasiopodomys fuscus as an important intermediate host for *Echinococcus multilocularis*: isolation and phylogenetic identification of the parasite. Infect Dis Poverty.

[23] Wang LY, Wang Q, Cai HX, Wang H, Huang Y, Feng Y (2021). Evaluation of fecal immunoassays for canine *Echinococcus* infection in China. PLoS Negl Trop Dis.

[24] Craig PS, Echinococcosis Working Group in China (2006). Epidemiology of human alveolar echinococcosis in China. Parasitol Int.

